# Structural Brain Lesions and Gait Pathology in Children With Spastic Cerebral Palsy

**DOI:** 10.3389/fnhum.2020.00275

**Published:** 2020-07-09

**Authors:** Eirini Papageorgiou, Nathalie De Beukelaer, Cristina Simon-Martinez, Lisa Mailleux, Anja Van Campenhout, Kaat Desloovere, Els Ortibus

**Affiliations:** ^1^Department of Rehabilitation Sciences, KU Leuven, Leuven, Belgium; ^2^Clinical Motion Analysis Laboratory, University Hospitals Leuven, Leuven, Belgium; ^3^Institute of Information Systems, University of Applied Sciences Western Switzerland (HES-SO), Sierre, Switzerland; ^4^Department of Development and Regeneration, KU Leuven, Leuven, Belgium; ^5^Department of Orthopedics, University Hospitals Leuven, Leuven, Belgium

**Keywords:** cerebral palsy, brain lesions, structural brain MRI, motor function, gait pathology

## Abstract

The interaction between brain damage and motor function is not yet fully understood in children with spastic cerebral palsy (CP). Therefore, a semi-quantitative MRI (sqMRI) scale was used to explore whether identified brain lesions related to functional abilities and gait pathology in this population. A retrospective cohort of ambulatory children with spastic CP was selected [*N* = 104; 52 bilateral (bCP) and 52 unilateral (uCP)]. Extent and location-specific scores were defined according to the sqMRI scale guidelines. The gross motor function classification system (GMFCS), the gait profile score (GPS), GPSs per motion plane, gait variable scores (GVS) and multiple-joint (MJ) gait patterns were related to brain lesion scores. In all groups, the global total brain scores correlated to the GPS (total: *r*_s_ = 0.404, *p* ≤ 0.001; bCP: *r*_s_ = 0.335, *p* ≤ 0.05; uCP: *r*_s_ = 0.493, *p* ≤ 0.001). The global total hemispheric scores correlated to the GMFCS (total: *r*_s_ = 0.392, *p* ≤ 0.001; bCP: *r*_s_ = 0.316, *p* ≤ 0.05; uCP: *r*_s_ = 0.331, *p* ≤ 0.05). The laterality scores of the hemispheres in the total group correlated negatively to the GMFCS level (*r*_s_ = −0.523, *p* ≤ 0.001) and the GVS-knee sagittal (*r*_s_ = −0.311, *p* ≤ 0.01). Lesion location, for the total group demonstrated positive correlations between parietal lobe involvement and the GPS (*r*_s_ = 0.321, *p* ≤ 0.001) and between periventricular layer damage and the GMFCS (*r*_s_ = 0.348, *p* ≤ 0.001). Involvement of the anterior part of the corpus callosum (CC) was associated with the GVS-hip sagittal in all groups (total: *r*_pb_ = 0.495, *p* ≤ 0.001; bCP: *r*_pb_ = 0.357, *p* ≤ 0.05; uCP: *r*_pb_ = 0.641, *p* ≤ 0.001). The global total hemispheric and laterality of the hemispheres scores differentiated between the minor and both the extension (*p* ≤ 0.001 and *p* ≤ 0.001) and flexion (*p* = 0.016 and *p* = 0.013, respectively) MJ patterns in the total group. Maximal periventricular involvement and CC intactness were associated with extension patterns (p ≤ 0.05 and p ≤ 0.001, respectively). Current findings demonstrated relationships between brain structure and motor function as well as pathological gait, in this cohort of children with CP. These results might facilitate the timely identification of gait pathology and, ultimately, guide individualized treatment planning of gait impairments in children with CP.

## Introduction

Children with cerebral palsy (CP) suffer from a non-progressive brain lesion that occurs in the developing fetal or infant brain (Rosenbaum et al., 2007). As a consequence, motor deficits (e.g., spasticity and muscle weakness) and functional disabilities (e.g., upper limb dysfunction and gait pathology) emerge during early childhood (Graham et al., 2016). The clinical severity of motor and functional impairments is frequently described by the topographic classification, i.e., bilateral (bCP) and unilateral (uCP) CP (Rosenbaum et al., 2007; Graham et al., 2016) and by the levels of the Gross Motor Function Classification System (GMFCS) (Palisano et al., 1997).

An increasing number of studies have explored the link between brain structure and functional impairments (Arnfield et al., 2013; Meyns et al., 2016; Mailleux et al., 2017a, b; Zhou et al., 2017; Cahill-rowley et al., 2019). Previous explorations in children with bCP, for example, have shown that increased damage to the total corpus callosum (CC) volume and increased lateral ventricle volume were associated with increased gait pathology (Meyns et al., 2016).

Neuro-imaging has played a substantial role in describing the pathogenesis and structure-function relationship in CP (Krägeloh-Mann and Horber, 2007; Himmelmann and Uvebrant, 2011). Because conventional magnetic resonance imaging (MRI) findings add considerable information to the early diagnosis of CP in children, harmonizing definitions is crucial (Novak et al., 2017). The Surveillance of Cerebral Palsy in Europe has suggested using the MRI Classification System (MRICS), where the predominant pathogenic lesion pattern is classified in broad categories, based on the presumed timing of the brain insult (Himmelmann et al., 2016). However, this classification system is restricted in providing a detailed neuro-anatomical characterization of the brain injury.

A reliable, semi-quantitative MRI (sqMRI) scale has recently been developed to assess both the extent and specific locations (e.g., layers, lobes, and subcortical structures) of the lesion. The scale is applicable in children from the age of 3 years onward, i.e., when myelination appears to be complete in both lobes and subcortical regions (Parazzini et al., 2002). In this scale, higher sqMRI scores indicate a higher brain involvement (Fiori et al., 2014). To date, this assessment, which has been developed specifically for CP, has shown correlations with communication in children with CP (Coleman et al., 2016; Laporta-Hoyos et al., 2018), upper limb sensorimotor outcomes and function in only uCP (Fiori et al., 2015; Pagnozzi et al., 2015; Mailleux et al., 2017a, b), as well as motor function and cognition in children with dyskinetic CP (Laporta-Hoyos et al., 2018). As a result, this semi-quantitative scale confirmed its potential to investigate structure–function relationships in children with CP after the age of 3 years (Fiori et al., 2014).

Nevertheless, the relationships between the sqMRI scale and general functional abilities of children with spastic CP and, more specifically, gait have not yet been investigated. This is crucial, since gait is one of the most frequent functional impairments in children with CP. Indeed, approximately 70% of children with CP are ambulatory (Ahlin et al., 2016). Gait in CP is commonly assessed with three-dimensional gait analysis (3DGA), which constitutes the “golden standard” (Wren et al., 2011; Meldrum et al., 2014). Gait indices, such as the Gait Profile score (GPS) (Baker et al., 2009), are commonly used outcome measures quantifying the overall degree of gait deviations, in comparison with gait data of typically developing (TD) peers. In addition, gait classification systems describe the walking patterns of children with CP and may be considered complementary to overall gait indices because they provide more insight regarding the direction of the observed deviation(s). The combination of gait indices and gait classification according to multiple joint (MJ) patterns (Papageorgiou et al., 2019a) may be key to comprehensively delineate gait pathology in children with CP and its relations to the brain lesion.

Children with bCP and uCP display differences in brain lesions (Krägeloh-Mann and Horber, 2007; Zhou et al., 2017), gross motor function (Himmelmann and Uvebrant, 2011; Holmes et al., 2018; Papageorgiou et al., 2019b), and gait (Meyns et al., 2016; Holmes et al., 2018). These population-based differences necessitate in-depth exploration of the possible interactions between the brain lesions of each group and their function or gait characteristics. Previous studies on the brain-gait relation in uCP and bCP included small samples (Meyns et al., 2016). Additionally, the combination of extent and location of the brain lesions has not been thoroughly investigated. Studies on the relationships between brain structure and functional disabilities of children with CP have used various methods, without comprehensive quantification of the respective pathologies. With respect to brain analysis, qualitative scales based on conventional MRI (Romei et al., 2007; Himmelmann and Uvebrant, 2011; MacFarlane et al., 2012; Taufika et al., 2012) and different neuroimaging modalities, such as diffusion tensor imaging (DTI) (Rose et al., 2007; Meyns et al., 2016; Cahill-rowley et al., 2019), have been applied. Regarding functional abilities and gait pathology, previous studies frequently used the gross motor function measure (Romei et al., 2007) and the GMFCS (Rose et al., 2007; Himmelmann and Uvebrant, 2011), gait patterns (MacFarlane et al., 2012; Taufika et al., 2012), spatiotemporal parameters (Cahill-rowley et al., 2019) or quantified outcome measures (Romei et al., 2007; Rose et al., 2007; Meyns et al., 2016; Cahill-rowley et al., 2019). The use of various brain and motor outcome measures might impede drawing overall conclusions concerning the structure–function relationship in children with CP. A deeper understanding of the underlying neural contributions could bridge the gap between the heterogeneous clinical presentations, as well as provide information and guidance for targeted, life-long treatments in children with CP (Rose et al., 2007; Graham et al., 2016; Zhou et al., 2017; Meyns et al., 2019).

Therefore, the aim of this retrospective study was to explore the relationships between the observed brain lesions in children with CP and their functional impairments with validated and easily obtainable tools, and with a special focus on gait pathology. This explorative study is the first to combine several previously explored, isolated parameters. The integration of several parameters is a new step towards a generalization of the findings. This methodological approach allows the comparison of the new findings with previous studies and the delineation of novel associations that may have been lost in those previous studies. Hence, we explored whether the extent and location of the brain lesions scored according to the sqMRI scale were related to a comprehensive assessment of (a) the functional abilities, using the GMFCS, and (b) gait pathology, by means of the GPS and MJ patterns, in children with CP. These relationships were further separately explored in bCP and uCP children.

## Materials and Methods

### Participants

A convenience sample was selected from the retrospective database of the Clinical Motion Analysis Laboratory of the University Hospitals Leuven, under the ethical approval provided by the Medical Ethical Committee of the University Hospitals Leuven (s56036). Permission to use and process retrospective patient data acquired during standard medical care was granted on condition that all patient information had been a priori anonymized and no patients would be included if they had requested so. Therefore, an informed written consent from all parents and/or patients was not acquired. This database comprised 3DGA sessions of more than 400 children, who were screened based on the following inclusion criteria: (a) a diagnosis of spastic bCP or uCP, (b) a 3DGA conducted between the ages of three and eight, (c) availability of a conventional brain MRI, acquired after the age of 3 years. Children were excluded if they showed marked clinical signs of dyskinesia or ataxia, if they had undergone any lower limb surgery in the past, more than three botulinum toxin type A (BoNT-A) treatments or if they had received a BoNT-A treatment less than 6 months before the 3DGA. These treatment criteria were applied in order to ensure homogeneity of the included sample, as well as to minimize the effect of treatment history on the analyses. In case multiple 3DGA sessions of the same patient fulfilled all criteria, the earliest one with sufficient data quality was chosen, to minimize the effect of treatment(s) or growth on gait. If multiple MRI scans were available, the first MRI after the age of 3 years and closest to the 3DGA was chosen. Additional data were collected and reported for all patients regarding commonly measured clinical impairments and comorbidities. Clinical impairments of the muscles acting in the sagittal plane were summed to form composite scores for spasticity, weakness, selectivity and passive range of motion (ROM) (Papageorgiou et al., 2019b). Comorbidities included visual, hearing and intellectual impairments, as well as a history of epilepsy. These were scored according to the guidelines of the Surveillance of CP in Europe (Cans et al., 2007).

### Brain MRI

Each MRI scan was scored by a trained pediatric neurologist (EO) who was blinded to patient characteristics and 3DGA results, and who was also involved in the original reliability study of the sqMRI (Fiori et al., 2014). Firstly, the MRICS was used to qualitatively classify all children’s brain lesions based on presumed lesion timing (Himmelmann et al., 2016). Only the predominant pattern based on the presumed timing of the lesion was reported for children who had multiple brain lesions, in accordance with the guidelines of the Surveillance of CP in Europe (Himmelmann et al., 2016). Children with a normal brain classification (i.e., category E) were not further scored with the sqMRI scale. The sqMRI scale is used to score the extent and location of the lesion and is preferably applied to the axial fluid-attenuated inversion recovery (FLAIR) sequences, which allow a clearer visualization of white matter lesions. If no FLAIR sequence was available for a patient, standard T1 sequences were used. The procedure was identical to that reported in the original publication, whereby the lesions were drawn on a paper template with six axial slices and scores were calculated based on the drawing (Fiori et al., 2014). Scoring brain lesions according to the sqMRI scale allows to score the extent and location of the lesion. In general, higher scores indicate larger damage, representing the extent of the brain lesion.

The *extent* of the brain lesion was expressed with the global total brain score (0–40), the global total hemispheric (0–24) and global total subcortical (0–10) scores, as well as the total CC and cerebellum scores (0–3, each). In addition to these, an adjusted global total brain score (0–37) was computed in case a sagittal MRI view was not available or was of poor quality, hindering the scoring of the CC. Finally, the laterality of the hemispheres was calculated based on the formula by Desmond et al. (1995), following the adaption by Coleman et al. (2016) (i.e., the difference between the two global hemispheric scores was divided by their sum, ranging between 0 and 1).

The *location* of the lesion was calculated based on detailed scores of the four brain lobes (i.e., frontal, parietal, temporal, and occipital; 0–3, each) in the most affected brain hemisphere. In cases of bilateral symmetrical lesions, the hemisphere that referred to the contralateral side of the most affected lower limb was used. Each lobe was further divided into three layers, namely periventricular, middle and cortico/subcortical (0–4, each). Lastly, all subcortical structures (the lenticular and caudate nuclei, the posterior limb of the internal capsule – PLIC, the thalamus and the brainstem) along with the individual parts of the CC (i.e., anterior, middle and posterior) were investigated in more detail (all scored as 0 or 1, indicating intactness or involvement, respectively).

### Gait Analysis

For children with uCP, only the lower limb that displayed neurological symptoms and motor deficits was taken into account. For children with bCP, the most spastic and/or weakest side was chosen based on the clinical records of each patient. This was decided in order to account for the interdependence between the motions of both affected sides, as well as for enhanced comparability between the two patient groups.

Standardized 3DGAs at a self-selected walking speed and in a barefoot condition were selected from the retrospective gait database for all included children. Kinematic, kinetic and electromyographic data were recorded at the time of the gait analysis, which was planned as a routine clinical assessment. However, only kinematic data were considered for the current study. The measurement system consisted of 10–15 optoelectronic cameras (Oxford Metrics, Oxford, United Kingdom) set up around a 10 m walkway. Markers were located on specific anatomical landmarks, according to the Vicon Plug-In-Gait model. Gait cycles were identified using the kinematic data as well as data from two force plates (Advanced Mechanical Technology Inc., United States) embedded in the walkway. After the identification of gait cycles, joint angles of the hip, knee and ankle, as well as segmental orientation of the pelvis and foot were calculated in Vicon Nexus software (Oxford Metrics, Oxford, United Kingdom). Next, all gait trials were imported into a custom-made Matlab® software (The MathWorks, Natick, MA, United States, 2015), to control for quality and any artifacts or potential outliers. To that end, the ROM and the knee varus-valgus angle values were taken into consideration as previously described (Schwartz et al., 2004). Outliers were defined by taking the average kinematic waveform of each lower limb as a reference and by inspecting the variability of the individual trials around the averaged waveform. A knee varus-valgus ROM ≥ 15°, a knee valgus angle ≤ −10° during swing phase or increased variability in comparison to the average kinematic waveform led to trial exclusion. Subsequently, all available, good-quality gait trials were averaged in this custom-made software, thus creating a new trial for each child. These new, averaged gait trials were used for all further analyses.

Firstly, the GMFCS was reported because it is a widely accepted, frequently reported and validated measure of gross motor function for children with CP (Palisano et al., 1997, 2000). Gait is one of the components of the GMFCS, with the levels I to III expressing the ability to walk, and higher level referring to severe gait impairments (Molloy et al., 2010; Hassani et al., 2011; Massaad et al., 2014; Õunpuu et al., 2015). Secondly, the GPS was calculated. The GPS is an overall gait index that summarizes all relevant kinematic deviations from gait kinematics of TD children. The TD database consisted of 23 children with a median age of 7 years, 2 months (range 4 years, 2 months – 7 years, 11 months) and no neurological or musculoskeletal disorders. The GPS constitutes the root mean square difference between the gait vector of each patient and that of TD peers and is expressed in degrees of motion (Baker et al., 2009). Apart from the overall GPS, a GPS per separate motion plane was calculated (i.e., GPS – sagittal/coronal/transverse), as well as individual gait variable scores (GVS) for nine relevant joint motions in the three motion planes (Baker et al., 2009). For the bCP children, laterality scores were also calculated for the four GPSs, based on the formula of Desmond et al. (1995). Even though the GPSs and GVSs represent the severity of overall gait deviations, the direction of these deviations remains unclear. To overcome this limitation, an additional gait measure was used, i.e., classification of the average gait trials according to a recently reported classification system of MJ gait patterns. These MJ gait patterns represent a series of combined motions at different lower limb joints accepted by the clinical CP community (Papageorgiou et al., 2019a). All assigned patterns were merged into three broad categories, indicating the general direction of the gait deviations. These three categories included: (i) ‘minor deviations,’ including the children displaying minor gait deviations and children classified with a drop foot, (ii) ‘extension patterns’, including children presenting with the genu recurvatum, true equinus or jump gait MJ patterns, and (iii) ‘flexion patterns,’ including children presenting with apparent equinus or crouch MJ patterns. A brief explanation of the GPSs, GVSs and MJ patterns’ classification can be found in Supplementary Figure 1.

### Statistical Analysis

All patient characteristics, brain lesion scores and gait scores were summarized and descriptive statistics were extracted. Normality of the data was not confirmed in all cases based on the Shapiro–Wilk test, hence non-parametric statistics were applied. Between-group comparisons were carried out with the Mann–Whitney *U* (MWU) test, as well as the Pearson chi-squared test (χ^2^) for the categorical outcomes, to identify whether the children with bCP and uCP showed baseline differences.

Due to the different types of study datasets, different statistical analyses were performed. A summary of all analyses can be found in Supplementary Table S1. The strength of all correlations was reported following the classification of Chan (2003). Correlation coefficients < 0.30 were classified as poor correlations and will not be discussed further. Coefficients between 0.30–0.50 and 0.50–0.80 were classified as fair and moderate correlations, respectively. Coefficients ≥ 0.80 were classified as very strong correlations (Chan, 2003).

Spearman’s rank correlations (*r*_s_) were performed to identify the relationships between the continuous or ordinal extent and location sqMRI scores and the GMFCS, the GPSs and the GVSs.

Point-biserial correlations (*r*_pb_) were used to explore the relationships between the normally distributed dichotomous location scores and gait pathology. The MWU test was applied to identify differences in the ranks of continuous GPSs and GVSs between the non-normally distributed dichotomous sqMRI categories.

Kruskal–Wallis comparisons were carried out to explore the differences in the continuous sqMRI scores across the three MJ patterns. If differences were identified, post-hoc MWU tests were performed.

Finally, the Pearson chi-squared test (χ^2^) was used to study the associations between the ordinal and dichotomous sqMRI scores and MJ patterns. The χ^2^ test was also applied for the dichotomous scores and their associations with the GMFCS. In case of significant associations, their strength was defined based on Cramer’s V, which depends on the degrees of freedom (DF) and was subsequently classified as weak, moderate or strong (Cohen, 1988). The interpretation rules of these values are described in Supplementary Table S1. The direction of these associations can be further examined with the adjusted standardized residuals (i.e., standardized residuals to control for the variations due to the sample size). The latter indicate which specific combinations of scores contribute more strongly to the identified associations. Adjusted standardized residuals follow a normal distribution [with ‘0’ as mean and ‘1’ representing one standard deviation (SD)]. Hence, values of adjusted standardized residuals larger than the mean +2 SDs indicate that two scores are observed more frequently together and are thus associated with each other. Similarly, values smaller than the mean −2 SDs indicate combinations that are statistically negatively associated with each other. All statistical analyses were performed in SPSS (IBM SPSS Statistics for Windows, version 24—IBM Corp., Armonk, NY, United States) with α = 0.05. Due to the exploratory nature of the present study, correction for multiple testing was only applied for the *post hoc* MWU analyses that were run after the Kruskal–Wallis comparisons (α = 0.017 – Šidák correction).

## Results

### Sample, Brain Lesion and Gait Characteristics

A total of 104 children with spastic CP fulfilled the inclusion criteria and were selected for study enrollment. Patient characteristics are summarized in Supplementary Table S2. At the time of MRI, the median age was 8 years, 5 months (range 3 years, 0 months – 17 years, 7 months) and the median age at which the 3DGA was performed was 5 years, 10 months (range 3 years, 7 months – 7 years, 10 months) with an average time of 2 years, 7 months between the two evaluations. The sample consisted of an equal number of children with bCP and uCP (*n* = 52). At the time of the gait analysis, 51% of the entire sample was naive to BoNT-A treatment, while less than 7% of the children had undergone three BoNT-A treatment sessions. In total, the sample consisted of children who were mildly affected by spasticity, weakness, impaired selectivity or contractures, as indicated by the median composite scores (e.g., 4 out of maximally 16 for spasticity). Moreover, most of the children had no reported associated comorbidities. Finally, all children received physical therapy (ranging from 1 to 6 sessions, with a median duration of 45 min per session). The majority of children (i.e., 81%) used ankle foot orthoses during the day, while 40% of the children additionally used night orthoses.

Brain lesion scores are summarized in Supplementary Table S3. A standard T1 sequence was used for only three children with no FLAIR sequence availability. Brain lesions classified following the MRICS showed that the vast majority of children had predominantly white matter injuries. Global total sqMRI scores presented a median score of 12 (interquartile range = 1.7–16.5) out of 40. Nine children (i.e., 8%) had a global total brain score of 20 or above, with the maximum score being 28.5 in one child. Adjusted global total brain scores were used for a total of 21 children. Of the remaining 83 children, 27 (33%) showed no corpus callosum involvement. Cerebellar involvement was even more uncommon, with 91% of the children having an intact cerebellum. As far as the lesion location scores of the most affected brain hemisphere are concerned, the periventricular layer displayed the highest damage (median = 3.5, interquartile range = 2–4), with 36% of the children having the maximum score of 4. With respect to the subcortical structures, almost 40% of the children had an involved PLIC and thalamus.

Gait parameters are summarized in Supplementary Table S4. Most of the children in the total group were highly functional, i.e., GMFCS level I (62%). The highest median scores were reported for the GPS-sagittal (8.69, interquartile range = 7.33 – 11.53) and the GVS-knee sagittal (12.19, interquartile range = 9.15 – 15.11). Most of the children (i.e., 48%) displayed an extension MJ pattern whereas 21 and 31% presented a minor and flexion MJ pattern, respectively.

### Relationships Between sqMRI Scores and Motor Function

**FIGURE 1:**
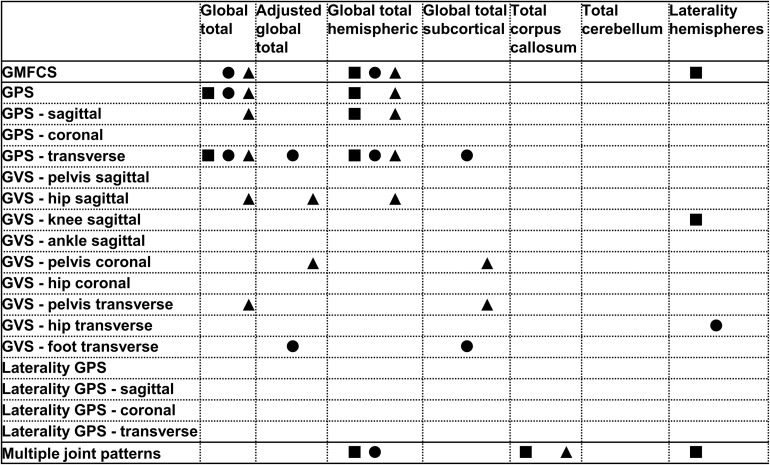
Summary of all statistically significant relationships identified between the brain lesion extent scores and the functional and gait scores (i.e., GMFCS, GPSs, GVSs, and MJ patterns). The total group is depicted with a square, the bCP with a circle and the uCP with a triangle (from left to right: total, bCP, uCP). GMFCS, gross motor function classification system; GPS, gait profile score; GVS, gait variable score; MJ, multiple joint; bCP, bilateral cerebral palsy; uCP, unilateral cerebral palsy.

**FIGURE 2:**
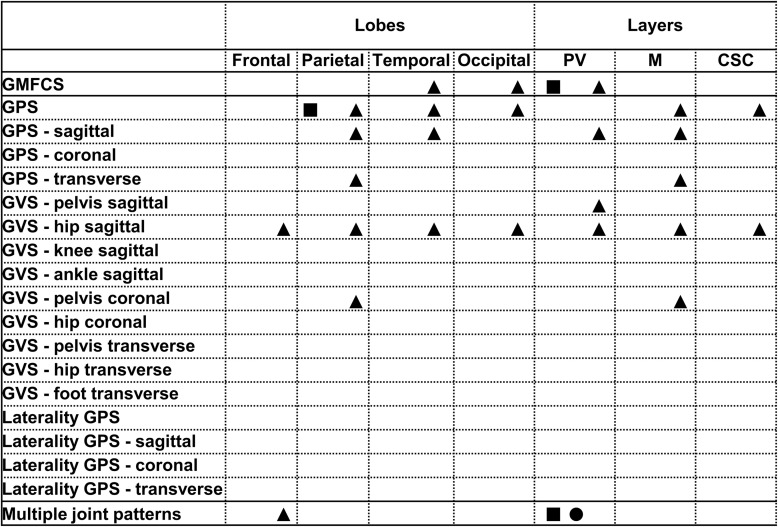
Summary of all statistically significant relationships identified between the lobar and layers brain lesion location scores and the functional and gait scores (i.e., GMFCS, GPSs, GVSs, and MJ patterns). The total group is depicted with a square, the bCP with a circle and the uCP with a triangle (from left to right: total, bCP, uCP). GMFCS, gross motor function classification system; GPS, gait profile score; GVS, gait variable score; MJ, multiple joint; bCP, bilateral cerebral palsy; uCP, unilateral cerebral palsy.

**FIGURE 3:**
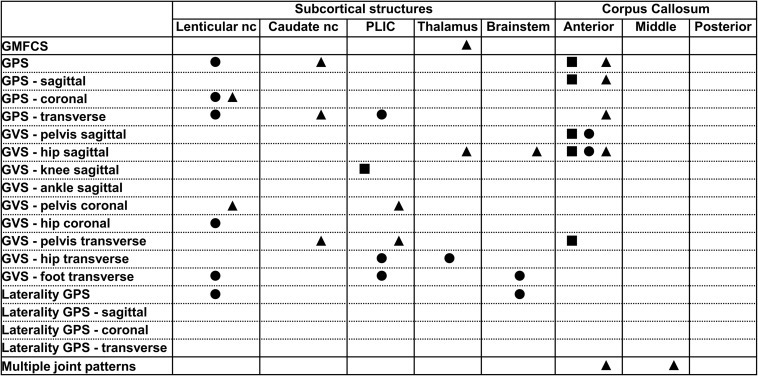
Summary of all statistically significant relationships identified between the dichotomous brain lesion location scores (i.e., subcortical structures and parts of the corpus callosum) and the functional and gait scores (i.e., GMFCS, GPSs, GVSs, and MJ patterns). The total group is depicted with a square, the bCP with a circle and the uCP with a triangle (from left to right: total, bCP, uCP). GMFCS, gross motor function classification system; GPS, gait profile score; GVS, gait variable score; MJ, multiple joint; bCP, bilateral cerebral palsy; uCP, unilateral cerebral palsy.

All relationships between the brain lesion scores and the GMFCS, GPS and its derivatives and the MJ patterns are summarized in Figures 1–3. In these figures, each statistically significant relationship that was identified across the various statistical analyses is represented with shapes for each group: i.e., square for the total, circle for the bCP and triangle for the uCP. Only the statistically significant results are reported in the tables, separately for each group (Tables 1–5). No relationships were established between the cerebellum and any of the motor function scores.

**TABLE 1:** Spearman’s rank correlations between the sqMRI extent scores and the scores of functional ability and gait for the total (*N* = 104), bilateral (*n* = 52), and unilateral (*n* = 52) groups.

	Global total^a^	Adjusted global total^b^	Global total hemispheric	Global total subcortical	Laterality hemispheres
**Total**					
GMFCS			0.392***		−0.523***
GPS	0.404***		0.387***		
GPS – sagittal			0.310***		
GPS – transverse	0.424***		0.372***		
GVS – knee sagittal					−0.311***
**bCP**					
GMFCS	0.325*		0.316*		
GPS	0.335*				
GPS – transverse	0.474**	0.566*	0.346*	0.370**	
GVS – hip transverse					−0.394**
GVS – foot transverse		0.595*		0.344*	
**uCP**					
GMFCS	0.336*		0.331*		
GPS	0.493***		0.422**		
GPS – sagittal	0.396**		0.379**		
GPS – transverse	0.339*		0.335*		
GVS – hip sagittal	0.469***	0.821*	0.485***		
GVS – pelvis coronal		0.786*		0.304*	
GVS – pelvis transverse	0.320*			0.308*	

*p ≤ 0.05; **p ≤ 0.01; ***p ≤ 0.001; sqMRI, semi-quantitative MRI scale [15]; GMFCS, gross motor function classification system; GPS, gait profile score; GVS, gait variable score; bCP, bilateral cerebral palsy; uCP, unilateral cerebral palsy; ^*a*^*n* = 83, 37, 46, respectively; ^*b*^global score when sagittal view MRI was missing, *n* = 21, 14, 7, respectively. Only the statistically significant results are shown.

**TABLE 2:** Spearman’s rank correlations between the sqMRI location scores of the most affected brain side and the scores of functional ability and gait for the total (*N* = 104) and unilateral (*n* = 52) groups.

	Lobes				Layers		
	Frontal	Parietal	Temporal	Occipital	PV	*M*	CSC
**Total**							
GMFCS					0.348***		
GPS		0.321***					
**uCP**							
GMFCS			0.312*	0.409**	0.364**		
GPS		0.450***	0.371**	0.305*		0.444***	0.338*
GPS – sagittal		0.357**	0.369**		0.312*	0.395**	
GPS – transverse		0.331*				0.309*	
GVS – pelvis sagittal					0.372**		
GVS – hip sagittal	0.340*	0.408**	0.438***	0.440***	0.512***	0.486***	0.315*
GVS – pelvis coronal		0.306*				0.316*	

*p ≤ 0.05; **p ≤ 0.01; ***p ≤ 0.001; sqMRI, semi-quantitative MRI scale [15]; PV, Periventricular; M, middle white matter; CSC, cortico/subcortical; uCP, unilateral cerebral palsy; GMFCS, gross motor function classification system; GPS, gait profile score; GVS, gait variable score. Only the statistically significant results are shown.

**TABLE 3:** Point-biserial correlations between (i) dichotomous sqMRI scores and (ii) continuous gait scores for the total (*N* = 104), bilateral (*n* = 52), and unilateral (*n* = 52) groups.

	Subcortical structures		Corpus callosum^a^
	Lenticular nucleus	Caudate nucleus	Anterior
**Total**			
GVS – hip sagittal			0.495***
**bCP**			
GPS	0.430***		
GVS – hip sagittal			0.357*
**uCP**			
GPS	0.449***		
GPS – sagittal			0.439**
GPS – transverse		0.426**	
GVS – hip sagittal			0.641***

*p ≤ 0.05; **p ≤ 0.01; ***p ≤ 0.001; sqMRI, semi-quantitative MRI scale [15]; PLIC, posterior limb of internal capsule; GVS, gait variable score; GPS, gait profile score; bCP, bilateral cerebral palsy; uCP, unilateral cerebral palsy; ^*a*^*n* = 83, 37, 46, respectively. Only the statistically significant results are shown.

**TABLE 4:** Statistically significant differences based on Mann–Whitney *U* test between the continuous gait scores for the total (*N* = 104), bilateral (*n* = 52), and unilateral (*n* = 52) groups for the dichotomous sqMRI scores.

	Subcortical structures					Corpus callosum^a^
	Lenticular nucleus	Caudate nucleus	PLIC	Thalamus	Brainstem	Anterior
**Total**						
GPS						0.009
GPS – sagittal						0.009
GVS – pelvis sagittal						0.032
GVS – knee sagittal			0.030^†^			
GVS – pelvis transverse						0.039
**bCP**						
GPS – coronal	0.043					
GPS – transverse	0.006		0.012			
GVS – pelvis sagittal						0.045
GVS – hip coronal	0.020					
GVS – hip transverse			0.048	0.046		
GVS – foot transverse	0.004		0.011		0.010	
Laterality GPS	0.039				0.008	
**uCP**						
GPS						0.006
GPS – coronal	0.031					
GPS – transverse						0.041
GVS – hip sagittal				0.028	0.017	
GVS – pelvis coronal	0.006		0.012			
GVS – pelvis transverse		0.041	0.014			

*sqMRI, semi-quantitative MRI scale [15]; PLIC, posterior limb of internal capsule; GPS, gait profile score; GVS, gait variable score; bCP, bilateral cerebral palsy; uCP, unilateral cerebral palsy; ^*a*^*n* = 83, 37, 46, respectively; ^†^an intact PLIC was related to a higher GVS – knee sagittal score. Only the statistically significant results are shown.*

**TABLE 5:** Statistically significant differences in sqMRI extent scores among the multiple joint patterns.

	Global total hemispheric	Laterality hemispheres
**Total**		
Kruskal-Wallis (p)	0.003	0.003
Minor vs. extension^a^	0.001	0.001
Minor vs. flexion^a^	0.016	0.013
Extension vs flexion^a^	NS	NS
**bCP**		
Kruskal–Wallis (p)	0.046	NS
Minor vs. extension^a^	NS	
Minor vs. flexion^a^	NS	
Extension vs flexion^a^	NS	

*sqMRI, semi-quantitative MRI scale [15]; NS, not significant; bCP, bilateral cerebral palsy; uCP, unilateral cerebral palsy; ^*a*^α = 0.017. Only the statistically significant results are shown.*

#### Brain Lesion Extent Scores in Relation to the GMFCS and Gait Pathology

All statistically significant correlations are reported in Table 1. No correlations were found between the total CC and either the GMFCS or gait pathology.

##### Total group

The global total sqMRI score fairly correlated to the GPS (*r*_s_ = 0.404, *p* ≤ 0.001) and the GPS-transverse (*r*_s_ = 0.424, *p* ≤ 0.001), indicating that more extensive brain damage corresponds to higher gait pathology. The global total hemispheric score correlated to the GMFCS (*r*_s_ = 0.392, *p* ≤ 0.001), GPS (*r*_s_ = 0.387, *p* ≤ 0.001), GPS-sagittal (*r*_s_ = 0.310, *p* ≤ 0.001), and GPS-transverse (*r*_s_ = 0.372, *p* ≤ 0.001). None of these global scores were related to the GVSs. In addition, no correlations were identified for the total group regarding the global total subcortical score. Finally, the laterality hemispheres’ score was negatively related to both the GMFCS level and the GVS-knee sagittal, indicating that children with more symmetric lesions, are classified in higher GMFCS levels and have more knee gait pathology, respectively.

##### Children with bCP

Fair correlations were identified between the global total sqMRI scores and the GMFCS, as well as the GPS (*r*_s_ = 0.325, *p* ≤ 0.05). The global total hemispheric score correlated fairly to the GMFCS (*r*_s_ = 0.316, *p* ≤ 0.05). In addition, all four global brain scores correlated to the GPS-transverse (i.e., global total – *r*_s_ = 0.474, *p* ≤ 0.01; adjusted global total – *r*_s_ = 0.566, *p* ≤ 0.05; global total hemispheric – *r*_s_ = 0.346, *p* ≤ 0.05; and global total subcortical scores - *r*_s_ = 0.370, *p* ≤ 0.01). Additional correlations were identified between the laterality of the hemispheres and the GVS-hip transverse (*r*_s_ = −0.394, *p* ≤ 0.01), as well as between both the adjusted global total score and the global total subcortical score and the GVS-foot transverse (*r*_s_ = 0.595, *p* ≤ 0.05; *r*_s_ = 0.344, *p* ≤ 0.05, respectively).

##### Children with uCP

The global total sqMRI and the global total hemispheric scores were fairly correlated with the GMFCS (*r*_s_ = 0.336, *p* ≤ 0.05; *r*_s_ = 0.331, *p* ≤ 0.05, respectively), and the GPS (*r*_s_ = 0.493, *p* ≤ 0.001; *r*_s_ = 0.422, *p* ≤ 0.01, respectively). Additional fair correlations were identified between both of these global brain scores and the GPS-sagittal, the GPS-transverse and the GVS-hip sagittal. In this group, relationships were found between all four global scores and proximal gait impairment scores (i.e., GVS-pelvis in the coronal and transverse planes and GVS-hip sagittal). The strongest relationships were reported between the adjusted global total score and both the GVS-hip sagittal and GVS-pelvis coronal (for *n* = 7: *r*_s_ = 0.821, *p* ≤ 0.05; *r*_s_ = 0.786, *p* ≤ 0.05, respectively).

#### Brain Lesion Location Scores in Relation to the GMFCS and Gait Pathology

All statistically significant results are reported in Tables 2–4. No relationships were found between the middle or the posterior parts of the CC and either the GMFCS or gait pathology.

##### Total group

The periventricular layer of the most affected brain hemisphere correlated fairly to the GMFCS (*r*_s_ = 0.348, *p* ≤ 0.001), as did the parietal lobe score with the GPS (*r*_s_ = 0.321, *p* ≤ 0.001) (Table 2). Furthermore, regarding the dichotomous variables of the sqMRI scale, the involvement of the anterior part of the CC was fairly correlated to the GVS-hip sagittal (*r*_pb_ = 0.495, *p* ≤ 0.001) (Table 3). Differences based on the MWU test demonstrated that the involvement of the anterior CC is related to increasing gait impairments (Table 4). In addition, an involved PLIC points to less severe pathology at the level of the knee in the sagittal plane (*p* = 0.03).

##### Children with bCP

No relationships were identified between the scores of the lobes or the layers and functional ability and gait in children with bCP (Table 2). As far as the dichotomous scores are concerned, two fair relationships were found, namely between (i) the lenticular nucleus and the GPS (*r*_pb_ = 0.430, *p* ≤ 0.001), and (ii) the anterior part of the CC and the GVS-hip sagittal (*r*_pb_ = 0.357, *p* ≤ 0.05) (Table 3). Differences based on the MWU test revealed that involvement of the lenticular nucleus, the PLIC, the thalamus, the brainstem and the anterior CC were associated with increasing gait pathology (Table 4).

##### Children with uCP

Positive, fair correlations were identified between the lobes and layers and the GMFCS, the GPSs and the GVSs (Table 2), indicating that increasing involvement of these lesion locations is observed with more pathological motor function. For example, the temporal and occipital lobes correlated to the GMFCS (*r*_s_ = 0.312, *p* ≤ 0.05; *r*_s_ = 0.409, *p* ≤ 0.01, respectively). Similarly, periventricular layer involvement was related to the GMFCS (*r*_s_ = 0.364, *p* ≤ 0.01). Moreover, the involvement in three lobes (i.e., parietal, temporal and occipital) and two layers (i.e., middle and cortico/subcortical) correlated to the GPS. Moderate correlations were identified between the periventricular layer score and the GVS-hip sagittal (*r*_s_ = 0.512, *p* ≤ 0.001).

With respect to the dichotomous lesion location scores, the GMFCS showed only one weak association with the thalamus in the uCP group (χ^2^ = 4.13, *p* ≤ 0.05, Cramer’s *V* = 0.282, DF = 1). More specifically, GMFCS level I was associated with an intact thalamus, while the opposite was the case for children with GMFCS level II (Supplementary Table S5). Moreover, lesions of the subcortical structures and the anterior part of the CC were related to increasing gait pathology (Tables 3, 4), with a moderate correlation between the anterior CC and GVS-hip sagittal (*r*_pb_ = 0.641, *p* ≤ 0.001), shown in Table 3.

#### Brain Lesion Scores in Relation to the MJ Patterns

**FIGURE 4:**
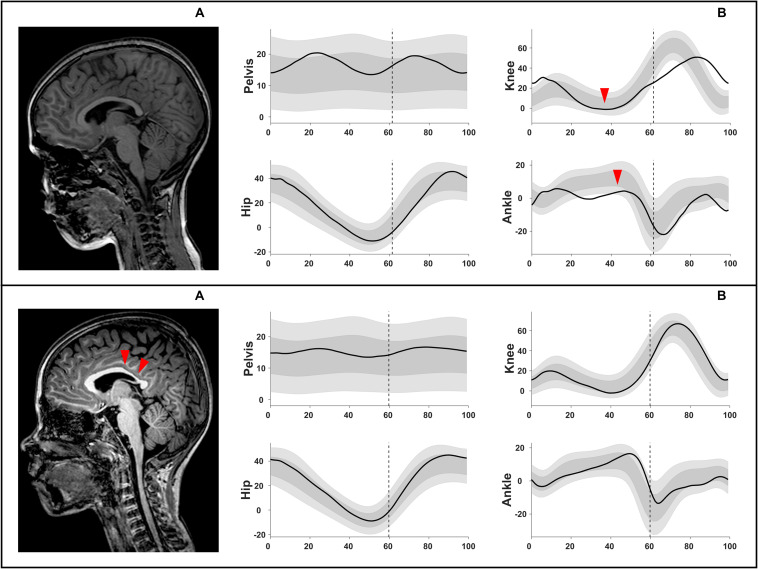
Example illustration of chi-squared analyses, shown in Supplementary Table S6. Upper row: **(A)** intact corpus callosum (score 0 out of 3) associated with **(B)** an extension MJ pattern (increased knee extension during midstance and reduced dorsiflexion). Lower row: **(A)** corpus callosum with score 2 out of 3 (involved middle and posterior parts) associated with **(B)** a minor deviations MJ pattern. MJ, multiple joint.

All statistically significant results are reported in Table 5, Figure 4, and Supplementary Tables S6, S7.

##### Total group

In the total group, significant differences in two of the global extent scores among the three MJ patterns were found (Table 5). The post-hoc MWU comparisons showed that significantly smaller global total hemispheric scores were found in the children with minor MJ patterns in comparison with the extension (*p* ≤ 0.001) and flexion (*p* = 0.016) MJ patterns. Additionally, higher laterality of the hemispheres scores were found in the minor deviations MJ patterns, compared to the extension (*p* ≤ 0.001) and flexion (*p* = 0.013) patterns.

The total CC was moderately associated with the MJ patterns (χ^2^ = 15.20, *p* ≤ 0.05, Cramer’s *V* = 0.303, DF = 2) (Figure 4 and Supplementary Table S6). An intact CC was associated with the extension MJ patterns, whereas an involvement of two parts (out of the three) of the CC was associated with the minor MJ patterns.

Furthermore, the periventricular layer was strongly associated with the MJ patterns of the total group (χ^2^ = 32.56, *p* ≤ 0.01, Cramer’s *V* = 0.396; DF = 2). This association further showed that a score of 2 was associated with the flexion patterns while the maximum involvement score of 4 was associated with the extension MJ patterns (Supplementary Table S7).

##### Children with bCP

Despite the observed differences in the global total hemispheric score among the MJ patterns (*p* = 0.046) none of the post-hoc comparisons was significant against the corrected α value (Table 5). Only the periventricular layer involvement was strongly associated with the MJ patterns (χ^2^ = 23.74, *p* ≤ 0.05, Cramer’s *V* = 0.478, DF = 2). A score of 1.5 (out of 4) was associated with minor MJ patterns, a score of 2 (out of 4) with flexion MJ patterns and total periventricular layer damage was associated with the extension patterns (Supplementary Table S7).

##### Children with uCP

The global extent scores were not associated with the MJ patterns in this group, with the exception of the total CC score (χ^2^ = 18.51, *p* ≤ 0.01, Cramer’s *V* = 0.454, DF = 2 – Supplementary Table S6). More specifically, an intact CC was associated with extension patterns, while a complete CC involvement was associated with flexion MJ patterns. Regarding the location scores, strong associations were found between the frontal lobe scores and the MJ patterns (χ^2^ = 25.09, *p* ≤ 0.05, Cramer’s *V* = 0.491, DF = 2 – Supplementary Table S7). A minimal frontal lobe involvement (i.e., score of 0.5 out of 3) as well as a higher involvement (i.e., score of 2.5 out of 3) were associated with minor MJ patterns while a score of 1 was associated with flexion patterns. Finally, a moderate association was found between the anterior CC score and MJ patterns (χ^2^ = 6.78, *p* ≤ 0.05, Cramer’s *V* = 0.388, DF = 1) and a strong association between the middle CC score and MJ patterns (χ^2^ = 12.47, *p* ≤ 0.01, Cramer’s *V* = 0.526, DF = 1). An intact middle CC was associated with extension patterns. Anterior and middle part involvement were associated with flexion patterns (Supplementary Table S7).

## Discussion

The need to understand the relationship between the underlying brain structure and motor function in children with CP is largely reflected in the breadth of studies over the last decade (Arnfield et al., 2013; Meyns et al., 2016; Mailleux et al., 2017a, b; Laporta-Hoyos et al., 2018). However, these studies have focused on upper limb deficits, toddlers with CP or children with dyskinetic CP, and the relationship between the brain lesion and functional impairments, such as gait, remains unknown, especially after the brain has reached a full myelination and the gait pattern has started to mature (Meyns et al., 2019). Therefore, the present study specifically aimed to investigate whether the extent and location of the brain lesion are related to measures of functional ability, as well as gait pathology, in children with spastic CP.

Differences in primary motor deficits (composite spasticity and passive ROM scores), as well as functional severity (i.e., GMFCS and gait) were found between the *two CP groups*, confirming that the children in the bCP group were more affected in comparison with the uCP group. When scored with the sqMRI scale, the structural damage of total hemispheres was larger in the bCP group, whereas the total subcortical and the laterality of the hemispheres scores were higher in the uCP group. For the latter, the lenticular, PLIC and brainstem were the most involved locations of the subcortical structures in the most affected brain side. The GMFCS levels were differently distributed among the two groups, with the children in the bCP group being less functional than those in the uCP group. All gait scores that were different between the two groups were significantly higher in children with bCP, representing a less impaired gait in children with uCP. Meyns et al. (2016) found the same trends of differences between bilateral and unilateral CP, even though they included fewer children in their study, with wider age ranges at the time of 3DGA and more uCP children with only unilateral brain lesions (4/25 versus 2/52 in the present study). In conclusion, our two groups reflected the expected differences in clinical presentation, extent, location and laterality of the lesion, as well as functional impairments (Fiori et al., 2015).

### Brain Structure – Motor Function Relationship

The links between brain structure and motor function were firstly investigated between the *brain lesion extent scores* and measures of *gross motor function*. The overall white matter damage (i.e., global total hemispheric score) fairly correlated to the GMFCS in all groups. However, the global total brain score in the total group did not correlate to the GMFCS. This is contradictory with recent findings in dyskinetic CP, where the global brain score was found to be a significant predictor for the GMFCS (Laporta-Hoyos et al., 2018). This difference might stem from several factors. In the present study, children of up to level III of the GMFCS were analyzed, whereas in the study of Laporta-Hoyos et al. (2018), one third of the included children had GMFCS levels IV or V. Furthermore, one third of the patients in this study only had lesions in the basal ganglia or thalamus (Laporta-Hoyos et al., 2018), while all children enrolled in the present study had additional lesions. An additional explanation might be that lower global brain scores were reported by Laporta-Hoyos et al. (2018), with 49% of the participants presenting with a total global brain score of ≤5.5 (out of 40) (Laporta-Hoyos et al., 2018). In the present study, only approximately 10% of the patients presented with such low global brain scores. Lastly, the total group in this study showed an equal distribution between bCP and uCP children, whereas the study of Laporta-Hoyos et al. (2018) included mostly bCP dyskinetic children (Laporta-Hoyos et al., 2018).

Interestingly, the laterality of the two hemispheres in the total group showed a moderately negative correlation to the GMFCS, suggesting that more symmetrical brain lesions were observed in less functional children. This could also mean that these children showed a decreased potential for plasticity, through a diminished ability to retain or rewire locomotor circuitry (Fiori et al., 2015). Future studies focusing on the different associations between brain lesions and motor function, and, based on the amount of laterality of the brain lesion, may shed more light on this hypothesis.

The *extent* of the brain lesion (i.e., global total score) was fairly positively related to *gait pathology* (total GPS) in all groups. Furthermore, both the global total and the global total hemispheric scores were fairly related to the GPS-transverse in all groups, while in the total group, only the laterality scores correlated to any of the GVSs. It should be noted that none of these significant correlations were strong ones. Additionally, no correlations were observed for the adjusted global total and the global total subcortical scores with gait in this group. The observed relations between *lesion extent* and gait appeared to be different between the *two CP* groups. For example, in the transverse plane, two brain extent scores (i.e., the adjusted global total and the global total subcortical scores) were related to the GVS of the foot in children with bCP (moderate and fair correlations, respectively), whereas only the global total brain score was fairly related to the GVS of the pelvis in children with uCP. In addition, in this group, the brain *extent scores* were mostly related to *gait pathology* of the proximal joints, resulting in fair to strong correlations.

Stronger correlations were observed in the uCP group compared to the bCP and total groups. These findings could be the result of a more homogenous cohort of uCP children, with 86% being categorized as GMFCS level I and 62% being naive to BoNT-A treatment. Moreover, the global total brain scores and global total hemispheric scores were mostly related to overall gait outcomes, i.e., the GPS and GPS-transverse. Similar results were obtained in a study of Romei et al. (2007), which showed that children with more severe brain injuries had more gait deviations in comparison with children with milder brain lesions (Romei et al., 2007). The associations between brain *extent scores* and the GPS in children with uCP were not observed in the findings of Meyns et al. (2016). However, differences between the current and the previous study might explain the disagreement in findings, including the use of a different neuro-imaging modality (i.e., standard MRI versus DTI) and the variability in the included sample characteristics (e.g., number of patients and age).

The *lesion location scores* of the total group showed fair correlations between the parietal lobe scores and the GPS, as well as between the periventricular layer and both the GMFCS. Based on the MRICS classification, most of the children in this study had a predominant white matter injury (73%). This distribution and the present findings are in line with an earlier population-based study (Himmelmann and Uvebrant, 2011). Furthermore, the associations for these specific *location scores* are in agreement with previous studies that reported on the role of the white matter regions in gait pathology (Cahill-rowley et al., 2019; Toda et al., 2019), even though no associations, emerged between the middle white matter or the cortico/subcortical layer and any of the functional measures in the total group.

Overall, the *lesion location* showed different correlations between the children with bCP and uCP. In the bCP group, only the subcortical structures, with the exception of the caudate nucleus, and the anterior part of the CC were associated with various gait scores (e.g., GVS-foot in the transverse plane). On the contrary, each of the lobar and layer scores, as well as all five subcortical structures’ scores, fairly to moderately correlated to either the GMFCS or at least one gait measure in uCP. Because previous research also revealed fair to strong relationships between subcortical scores (i.e., PLIC and thalamus) and upper limb motor function in children with uCP (Mailleux et al., 2017a), the present findings further suggest a role of these *lesion locations* in the functionality of children with uCP.

MJ patterns were used in order to explore whether brain lesion scores differed according to the direction of the gait deviations. The global total hemispheric and laterality of the hemispheres scores indicated that the minor MJ patterns are different from both the extension and the flexion MJ patterns. Nonetheless, these *lesion extent scores* were not different between the more pathological MJ patterns. On the other hand, based on the total CC scores a differentiation between the extension and flexion MJ patterns in the total group was possible. Specifically, an intact total CC was moderately and positively associated with the extension MJ patterns and negatively associated with the flexion MJ patterns. Moreover, the *lesion location* scores additionally showed some differences between the two more pathological patterns. In the total and bCP groups, a score of 2 (out of 4) assigned in the periventricular layer was observed in children with flexion patterns while a fully involved periventricular layer was associated with extension patterns.

In the uCP group, strong positive associations were identified between an intact total CC and extension MJ patterns, but also between a fully involved total CC (score 3 out of 3) and flexion MJ patterns. Furthermore, an involved anterior or middle part of the CC was observed with flexion MJ patterns, whilst an intact middle part was related to extension patterns. Based on imaging studies, the anterior CC part seems important for actual and imagined walking, since premotor tracts are running through this location (Jahn et al., 2004; Bakker et al., 2008; la Fougère et al., 2010). The current results supported the emerging interest to investigate the CC and are in line with previous studies, where the anterior part of the CC was found to be significantly correlated to various gait metrics in toddlers (Rose et al., 2015; Cahill-rowley et al., 2019) or older children (Meyns et al., 2016). Those studies, however, have used more advanced imaging techniques (i.e., DTI). Hence, future investigations could explore the relationship of the CC –or its parts- to the GMFCS and gait patterns with more advanced measurements, such as measuring CC volume.

In summary, in the total group, the minor MJ patterns were associated with lower global total hemispheric and higher laterality scores, as well as an involvement of two CC parts and a score of 1.5 (out of 4) in the periventricular layer. The extension and flexion MJ patterns showed higher global total hemispheric and lower laterality scores in comparison with the minor MJ patterns. Additionally, for the extension MJ patterns, intactness of the total CC and maximal periventricular layer involvement were found. Lastly, the flexion MJ patterns were strongly positively associated with a score of 2 in the periventricular layer and were moderately negatively associated with an intact total CC.

### Limitations and Future Directions

This study has some limitations related to brain and gait metrics. First, the vast heterogeneity in the clinical picture of CP poses numerous challenges. Unraveling the relationships between the structural lesions and functional impairments is not straightforward. Therefore, a sample as homogenous as possible was selected. MRI scans performed after the age of 3 years were included, as suggested in the protocol of Fiori et al. (2014). At that age, the brain lesion has reached quite a mature level, indicating that the lesion is more stable in comparison with the neonatal brain state (Parazzini et al., 2002; Hermoye et al., 2006; Welker and Patton, 2012). During the neonatal period, lesions might be incorrectly classified as normal, because periventricular lesions or lesions to the gray matter could remain undetected (Himmelmann et al., 2016). Previous research indicated that the myelination process is finished in both the lobes and subcortical regions by the age of 3 years (Parazzini et al., 2002), thus enabling assessment of white matter lesions. Even though brain maturation may still be ongoing in other regions, this study focused on presumably stable brain lesions. The children included in this study had undergone a 3DGA at a young age, namely after the age of three, but before the growth spurt or the development of severe secondary deformities. This age range restriction resulted in the inclusion of children who had not received neuro- or orthopedic surgery or were minimally treated with BoNT-A, further ensuring a homogeneous sample. Whether this minimal treatment history has an impact on the observed relationships remains to be explored.

Moreover, children are considered to have at least started to obtain a stable gait pattern around that age (Sutherland, 1997), with a mature gait pattern emerging around the age of 5 years (Simon et al., 1978). The current findings may be affected by the dynamic nature of gait in children with. Young children may be characterized by inconsistency between repeated gait trials, suggesting an immature gait pattern. However, at the time of the 3DGA, 86% of the children had already been ambulant for at least 24 months, ensuring sufficient maturation of their gait pattern. For the remaining 14%, who had only been ambulant for more than 12 months, the use of averaged gait data filters this inconsistency between the gait trials. It is important to highlight that the included study sample covers the current natural history of growing children with CP. Future studies with longitudinal data and large study samples may further explore to what extent changes in gait pathology throughout growth may influence the associations between brain lesions and motor outcomes. The choice of age ranges was necessary to ensure homogeneity of the included sample. Furthermore, even younger children have been included in previous studies investigating the relationships between brain lesions and gait metrics (Rose et al., 2015; Cahill-rowley et al., 2019). All studies have aspired to clarify these relationships, in light of timely and appropriate treatment administration for children with CP. Due to the retrospective nature of this study and based on data availability, there was a substantial gap between the age at MRI and 3DGA. The median difference was 2 years, 7 months, with the MRIs having mostly been acquired at an older age. Assuming that the studied brain lesions are static, this difference in acquisition timings should not have influenced the current results.

Neuroimaging has established its role in the diagnostic process of CP, with brain MRI being increasingly accessible to clinicians (Novak et al., 2017). However, other imaging modalities, such as DTI, have advantages over the use and interpretation of structural brain MRI. These include higher sensitivity to identify white matter lesions (Hoon and Faria, 2010) or the involvement of certain locations, e.g., the volume of the CC. The use of conventional MRI could explain the diverse results in comparison with Meyns et al. (2016). MRI based on DTI, however, is not routinely available or used in clinical settings, rather (still) mostly applied for research purposes (Sutherland, 1997). Given that knowledge of the functional and gait characteristics is essential for clinical decision-making and treatment planning, the aim of our study was to provide insights in the neural correlates via a standardized and detailed scale using conventional neuro-imaging methods. The current findings delineated fair to strong associations between the brain lesions and motor function, based on conventional structural MRI, which is more clinically accessible and less time-consuming. The automation of the sqMRI brain scale could be a promising future direction to facilitate and optimize the transfer to clinical practice (Pagnozzi et al., 2015).

This exploratory study did not establish predictions of functional abilities and gait pathology based on the *extent and location* of the investigated brain lesions, nor did it apply statistical corrections for multiple testing. This study is a first step in providing a comprehensive quantification of the relationships of brain lesions with functional abilities and gait pathology in children with CP, using an extensive set of outcome parameters. Moreover, prediction analyses would require larger sample sizes, since the brain-gait interaction is considered obscure. Indeed, additional to neural injury, other factors are also suspected to be the causes of gait pathology in children with CP, such as the combination of the effect of spasticity, muscle weakness, and other motor deficits, as well as aberrant growth or compensation strategies (Baker et al., 2016). Future analyses can apply different statistics (e.g., logistic or linear regression models) on larger sample sizes, in order to accommodate for all different variable types within the sqMRI scale and the various motor function measures. The ultimate goal is to identify whether motor function and gait pathology can timely be explained and guided based on the underlying neurological correlates. Based on the current study results, preliminary treatment guidance can be suggested, in particular with respect to MJ patterns. Such clinical reasoning could, for example, be based on the moderate to strong associations of the intact total CC and full periventricular layer involvement with extension MJ patterns. Should these associations be validated by future studies, clinicians could promote adequate ankle dorsiflexion or limit knee hyperextension by adapting the physiotherapeutic exercise program accordingly, by providing properly tuned orthoses that prevent hyperextension, or by providing stimulating activities that facilitate the desired motions. Nevertheless, caution is needed before advising targeted and individualized interventions before the age of 3 years, based on the current findings.

## Conclusion

In general, this is the first study that used the sqMRI scale to investigate the relationships between, on the one hand, the *brain lesion extent and location* and, on the other hand, *motor function*, with a focus on *pathological gait*. A comprehensive gait assessment was studied, including not only the GPSs and the GVSs, but also MJ patterns. The preliminary study findings seem promising, yet caution is warranted when interpreting these results due to the exploratory nature of this study. Nevertheless, the analyses based on the GMFCS as well as the GPSs and GVSs demonstrated that children with more extensive brain lesions have increased gait pathology, and differences in brain lesion scores were found among the MJ gait patterns. This study focused not only on a generic sample of children with spastic CP but also included two separate patient groups based on topographic classification. These additional analyses revealed more relations between the *lesion extent and location* and the *gait pathology* in children with uCP, suggesting that it may be especially interesting to further investigate the specific *locations* of the brain injury firstly in children with uCP. In addition, this study identified the neuro-anatomical characterization of the brain injury and gait pathology after the age of 3 years, suggesting some guidelines of treatment planning in children with mature brain lesions and stable gait patterns. Finally, this exploratory study confirmed the existence of a structure – function relationship, with a focus on pathological gait, in children with spastic CP which has not been extensively documented in the past.

## Data Availability Statement

The datasets generated for this study are available on request to the corresponding author.

## Ethics Statement

The studies involving human participants were reviewed and approved by Medical Ethical Committee of the University hospitals Leuven (s56036). Written informed consent from the participants’ legal guardian/next of kin was not required to participate in this study in accordance with the national legislation and the institutional requirements.

## Author Contributions

EP, ND, KD, and EO was designed the study. EP and ND were responsible for gait data acquisition. EO was responsible for brain MRI scoring. EP and ND conducted the all presented analyses. All the authors had complete access to the study data throughout the study, contributed to the interpretation of the results, involved in the critical revision and editing of the manuscript, which was written equally by EP and ND and are co-first authors, approved the final version of the manuscript and agree to be accountable for the content of the work.

## Conflict of Interest

The authors declare that the research was conducted in the absence of any commercial or financial relationships that could be construed as a potential conflict of interest.
