# Supplementary material for: Structural Brain Lesions and Gait Pathology in Children With Spastic Cerebral Palsy
Source: Front Hum Neurosci. 2020 Jul 9;14:275. doi: 10.3389/fnhum.2020.00275 (PMC7363943; doi:10.3389/fnhum.2020.00275)
Supplement: Supplementary file 1 [file Table_1.pdf]

Table S1. Summary of all performed analyses among the various brain lesion and motor function measures.

Analysis	sqMRI scores	Function	Gait	Reported results
**1. Spearman's rank correlations** (r_s)	Continuous<br>(Global total; adjusted global total; global total hemispheric; global total subcortical; laterality hemispheres)	Ordinal (GMFCS)	Continuous (GPSs and GVSs)	**Correlation coefficients and p-values**
	Ordinal<br>(Total corpus callosum; total cerebellum; lobes: frontal, parietal, temporal, occipital; layers: PV, M, CSC)			
**2. Point-biserial correlations**[a] (r_{pb})	Dichotomous<br>(Subcortical structures: lenticular nucleus, caudate nucleus, PLIC, thalamus, brainstem;<br>Corpus callosum: anterior, middle, posterior)		Continuous (GPSs and GVSs)	**Correlation coefficients and p-values**
**3. Mann-Whitney U**	Dichotomous<br>(Subcortical structures: lenticular nucleus, caudate nucleus, PLIC, thalamus, brainstem;<br>Corpus callosum: anterior, middle, posterior)		Continuous (GPSs and GVSs)	**p-values**
**4. Kruskal-Wallis (with post-hoc Mann-Whitney U)**[b]	Continuous<br>(Global total; adjusted global total; global total hemispheric; global total subcortical; laterality hemispheres)		Nominal (MJ patterns)	**p-values**
**5. Pearson chi-squared**	Ordinal<br>(Total corpus callosum; total cerebellum; lobes: frontal, parietal, temporal, occipital; layers: PV, M, CSC)		Nominal (MJ patterns)	**χ^2, p-values and Cramer's V, interpreted based on DF**<br><br>For DF=1:<br>i) Weak association if $0.10<V<0.30$<br>ii) Moderate association if $0.30<V<0.50$<br>iii) Strong associations if $V>0.50$<br>For DF=2:<br>i)Weak association if $0.07<V<0.21$<br>ii) Moderate association if $0.21<V<0.35$<br>iii) Strong associations if $V>0.35$
	Dichotomous<br>(Subcortical structures: lenticular nucleus, caudate nucleus, PLIC, thalamus, brainstem;<br>Corpus callosum: anterior, middle, posterior)	Ordinal (GMFCS)		

sqMRI, semi-quantitative MRI scale [1]; PV, Periventricular; M, middle white matter; CSC, cortico/subcortical; PLIC, posterior limb of internal capsule; GMFCS, gross motor function classification system; GPS, gait profile score; GVS, gait variable score; MJ, multiple-joint; DF, degrees of freedom [2]; [a] the GPSs and GVSs within each 0 or 1 score had to be normally distributed, when normality was not met, a Mann-Whitney U test was performed; [b] $\alpha = 0.017$.

Table S2. General patients' characteristics and between group comparisons.

		Total (N=104)	bCP (n=52)	uCP (n=52)	p (MWU)	p (χ^2)
**Age at 3DGA [years]**	Median (IQR)	5.89 (5.09 - 6.88)	5.64 (5.02 - 6.68)	6.01 (5.29 - 7.11)	0.109	
**Age at MRI [years]**	Median (IQR)	8.38 (5.44 - 10.79)	6.78 (5.44 - 9.70)	9.79 (5.67 - 11.84)	0.015*	
**Gender**						0.689
	Boys [n (%)]	62 (59.6%)	32 (61.5%)	30 (57.7%)		
	Girls [n (%)]	42 (40.4%)	20 (38.5%)	22 (42.3%)		
**Weight (kg)**	Median (IQR)	18.85 (16.73 - 21.83)	17.9 (16.05 - 20.33)	20.55 (18.05 - 23.27)	≤0.001*	
**Height (m)**	Median (IQR)	1.14 (1.06 - 1.21)	1.12 (1.04 - 1.17)	1.17 (1.09 - 1.23)	0.004*	
**BoNT-A**						0.005*
	0 [n (%)]	53 (51%)	21 (40.4%)	32 (61.5%)		
	1 [n (%)]	23 (22.1%)	9 (17.3%)	14 (26.9%)		
	2 [n (%)]	21 (20.2%)	16 (30.8%)	5 (9.7%)		
	3 [n (%)]	7 (6.7%)	6 (11.5%)	1 (1.9%)		
**Composite spasticity[a] (0-16)**	Median (IQR)	4 (3 - 5.5)	5.25 (3.5 - 6.5)	3.5 (2.5 - 4.5)	≤0.001*	
**Composite weakness[a] (0-30)**	Median (IQR)	22 (20 - 23)[b]	21.5 (19 - 23)[c]	22 (20 - 24)[d]	0.132	
**Composite selectivity[a] (0-12)**	Median (IQR)	10.5 (8.5 - 11.25)[b]	9.5 (8.5 - 11)[c]	10.5 (9 - 11.5)[d]	0.405	
**Composite passive ROM[a] (0-6)**	Median (IQR)	2 (1 - 3)	2.5 (1 - 4)	1 (1 - 2)	0.007*	
**Comorbidities**						
	Visual impairment					0.018*
	Yes [n (%)]	48 (46.2%)	30 (57.7%)	18 (34.6%)		
	No [n (%)]	56 (53.8%)	22 (42.3%)	34 (65.4%)		
	Hearing impairment					1.000
	Yes [n (%)]	8 (7.7%)	4 (7.7%)	4 (7.7%)		
	No [n (%)]	96 (92.3%)	48 (92.3%)	48 (92.3%)		
	Intellectual impairment					0.139
	Yes [n (%)]	11 (10.6%)	7 (13.5%)	4 (7.7%)		
	No [n (%)]	58 (55.8%)	24 (46.1%)	34 (65.4%)		
	Unknown [n (%)]	35 (33.6%)	21 (40.4%)	14 (26.9%)		
	Epilepsy					0.222
	Yes [n (%)]	21 (20.2%)	8 (15.4%)	13 (25%)		
	No [n (%)]	83 (79.8%)	44 (84.6%)	39 (75%)		
**Physical therapy**						1.000
	Yes [n (%)]	100 (100%)	100 (100%)	100 (100%)		
	No [n (%)]	0	0	0		
**Sessions per week**	Median (IQR)	3 (2 - 3)	3 (2 - 3,8)	2 (2 - 3)	0.043*	
**Duration (in minutes)**	Median (IQR)	45 (30 - 60)	60 (30 - 60)	30 (30 - 60)	0.028*	
**Day ortheses**						0.135
	Yes [n (%)]	84 (80.8%)	39 (75%)	45 (86.5%)		
	No [n (%)]	20 (19.2%)	13 (25%)	7 (13.5%)		
**Night Ortheses**						0.689
	Yes [n (%)]	42 (40.4%)	22 (42.3%)	20 (38.5%)		
	No [n (%)]	62 (59.6%)	30 (57.7%)	32 (61.5%)		

*$p < 0.05$; bCP, bilateral cerebral palsy; uCP, unilateral cerebral palsy; MWU, Mann-Whitney U test; χ^2, Pearson chi squared; 3DGA, three-dimensional gait analysis; IQR, interquartile range; MRI, magnetic resonance imaging; BoNT-A, botulinum toxin type A treatments; ROM, range of motion; [a]composite scores are defined based on [3]; [b]n=69; [c]n=31; [d]n=38.

Table S3. Brain lesion scores and between group comparisons.

		Total (N=104)	bCP (n=52)	uCP (n=52)	p (MWU)	p (χ^2)
**MRICS**						0.126
	Maldevelopment [n (%)]	2 (1.9%)	1 (1.9%)	1 (1.9%)		
	Predominant white matter injury [n (%)]	76 (73.1%)	42 (80.8%)	34 (65.4%)		
	Predominant grey matter injury [n (%)]	17 (16.3%)	4 (7.7%)	13 (25%)		
	Miscellaneous changes [n (%)]	9 (8.7%)	5 (9.6%)	4 (7.7%)		
**Lesion extent scores**						
Global (0-40)	Median (IQR)	12 (7.5 - 16.5)[b]	13 (7.38 - 16.13)[c]	11.5 (7.75 - 17)[d]	0.721	
Adjusted global (0-37)[a]	Median (IQR)	11 (7.25 - 15)[e]	13.5 (7.88 - 15.75)[f]	10 (4.5 - 11)[g]	0.079	
Total hemispheric (0-24)	Median (IQR)	9.5 (5.5 - 12.5)	10 (6.63 - 12.88)	7.5 (4.5 - 11.88)	0.015*	
Total subcortical (0-10)	Median (IQR)	1 (0 - 3)	0 (0 - 2)	2 (0 - 4)	≤0.001*	
Corpus callosum (0-3)	Median (IQR)	1 (0 - 2)[b]	1 (0 - 2)[c]	1 (0 - 2)[d]		0.802
Cerebellum (0-3)	Median (IQR)	0 (0 - 0)	0 (0 - 0)	0 (0 - 0)		0.530
Laterality hemispheres	Median (IQR)	0.11 (0.04 - 0.63)	0.04 (0.0 - 0.08)	0.62 (0.19 - 1)	≤0.001*	
**Lesion location scores of the most affected brain side**						
Frontal lobe (0-3)	Median (IQR)	1.5 (1 - 1.5)	1.5 (1 - 1.5)	1.25 (1 - 2)		0.008*
Parietal lobe (0-3)	Median (IQR)	1.5 (1.5 - 2)	1.5 (1.5 - 2)	1.5 (1.13 - 2.88)		0.072
Temporal lobe (0-3)	Median (IQR)	1.5 (0.5 - 2)	1.5 (0.5 - 1.88)	1.5 (0.5 - 2)		0.111
Occipital lobe (0-3)	Median (IQR)	1.5 (0.5 - 2)	1.5 (0.5 - 2)	1.5 (0.5 - 2)		0.068
PV layer (0-4)	Median (IQR)	3.5 (2 - 4)	3.5 (2.63 - 4)	3 (2 - 4)		0.742
M layer (0-4)	Median (IQR)	2 (1.5 - 3)	2 (1.5 - 2.88)	2 (1.5 - 3)		0.599
CSC layer (0-4)	Median (IQR)	0 (0 - 0.5)	0 (0 - 0)	0 (0 - 2.38)		0.191
Lenticular nucleus (0-1)						≤0.001*
	Intact [n (%)]	84 (80.8%)	49 (94.2%)	34 (65.4%)		
	Involved [n (%)]	20 (19.2%)	3 (5.8%)	18 (34.6%)		
Caudate nucleus (0-1)						0.402
	Intact [n (%)]	90 (86.5%)	46 (88.5%)	43 (82.7%)		
	Involved [n (%)]	14 (13.5%)	6 (11.5%)	9 (17.3%)		
PLIC (0-1)						≤0.001*
	Intact [n (%)]	63 (60.6%)	42 (80.8%)	20 (38.5%)		
	Involved [n (%)]	41 (39.4%)	10 (19.2%)	32 (61.5%)		
Thalamus (0-1)						0.073
	Intact [n (%)]	63 (60.6%)	35 (67.3%)	26 (50%)		
	Involved [n (%)]	41 (39.4%)	17 (32.7%)	26 (50%)		
Brainstem (0-1)						≤0.001*
	Intact [n (%)]	73 (70.2%)	48 (92.3%)	24 (46.2%)		
	Involved [n (%)]	31 (29.8%)	4 (7.7%)	28 (53.8%)		
Anterior part of corpus callosum (0-1)						0.898
	Intact [n (%)]	65 (78.3%)[b]	30 (57.7%)[c]	35 (67.3%)[d]		
	Involved [n (%)]	18 (21.7%)[b]	8 (15.4%)[c]	10 (19.2%)[d]		
Middle part of corpus callosum (0-1)						0.734
	Intact [n (%)]	41 (49.4%)[b]	18 (34.6%)[c]	23 (44.2%)[d]		
	Involved [n (%)]	42 (50.6%)[b]	20 (38.5%)[c]	22 (42.3%)[d]		
Posterior part of corpus callosum (0-1)						0.703
	Intact [n (%)]	28 (33.7%)[b]	12 (23.1%)[c]	16 (30.8%)[d]		
	Involved [n (%)]	55 (66.3%)[b]	26 (50%)[c]	29 (55.7%)[d]		

*$p < 0.05$; bCP, bilateral cerebral palsy; uCP, unilateral cerebral palsy; MWU, Mann-Whitney U test; χ^2, Pearson chi squared; MRICS, magnetic resonance imaging classification system [4]; IQR, interquartile range; PLIC, posterior limb of internal capsule; PV, periventricular; M, middle white matter; CSC, cortico/subcortical; [a]global score when sagittal view MRI was missing; [b]n=83; [c]n=38; [d]n=45; [e]n=21; [f]n=14; [g]n=7.

Table S4. Gait scores and between group comparisons.

		**Total (N=104)**	**bCP (n=51)**	**uCP (n=53)**	**p (MWU)**	**p (χ^2)**
**GMFCS**						≤0.001*
	I [n (%)]	64 (61.5%)	19 (36.6%)	45 (86.5%)		
	II [n (%)]	30 (28.9%)	23 (44.2%)	7 (13.5%)		
	III [n (%)]	10 (9.6%)	10 (19.2%)	0 (0%)		
GPS	Median (IQR)	8.03 (6.64 - 9.67)	9.10 (7.24 - 11.46)	7.32 (6.17 - 8.52)	≤0.001*	
GPS - sagittal	Median (IQR)	8.96 (7.33 - 11.53)	10.22 (8.34 - 12.83)	8.05 (6.52 - 9.52)	≤0.001*	
GPS - coronal	Median (IQR)	3.58 (2.74 - 5.11)	3.77 (3.08 - 5.27)	3.39 (2.21 - 4.48)	0.059	
GPS - transverse	Median (IQR)	7.67 (5.92 - 11.30)	7.97 (6.16 - 12.74)	7.40 (5.83 - 10.21)	0.214	
GVS - pelvis sagittal	Median (IQR)	4.79 (2.93 - 7.40)	5.76 (3.14 - 9.51)	4.42 (2.82 - 6.26)	0.036*	
GVS - hip sagittal	Median (IQR)	6.82 (5.13 - 10.47)	8.53 (5.61 - 11.02)	6.09 (5.06 - 9.01)	0.036*	
GVS - knee sagittal	Median (IQR)	12.19 (9.15 - 15.11)	14.21 (10.69 - 17.75)	10.55 (7.46 - 12.76)	≤0.001*	
GVS - ankle sagittal	Median (IQR)	7.83 (6.03 - 10.52)	8.62 (6.22 - 11.78)	7.41 (5.88 - 9.39)	0.123	
GVS - pelvis coronal	Median (IQR)	2.56 (1.71 - 4.07)	3.17 (1.99 - 4.53)	2.10 (1.61 - 3.76)	0.012*	
GVS - hip coronal	Median (IQR)	4.17 (3.08 - 5.67)	4.18 (3.26 - 5.71)	4.16 (2.38 - 5.47)	0.205	
GVS - pelvis transverse	Median (IQR)	5.24 (3.54 - 8.05)	5.64 (3.35 - 8.64)	5.09 (3.59 - 7.56)	0.585	
GVS - hip transverse	Median (IQR)	7.45 (5.13 - 11.03)	7.50 (5.35 - 11.34)	7.45 (4.92 - 10.41)	0.876	
GVS - foot transverse	Median (IQR)	7.84 (4.44 - 13.98)	8.25 (4.54 - 16.58)	6.95 (4.13 - 11.85)	0.269	
Laterality GPS	Median (IQR)		0.07 (0.03 - 0.1)			
Laterality GPS - sagittal	Median (IQR)		0.05 (0.02 - 0.1)			
Laterality GPS - coronal	Median (IQR)		0.11 (0.07 - 0.21)			
Laterality GPS - transverse	Median (IQR)		0.13 (0.05 - 0.22)			
**Multiple joint patterns**						0.038*
	Minor [n (%)]	22 (21.1%)	6 (11.5%)	16 (30.8%)		
	Extension [n (%)]	50 (48.1%)	30 (57.7%)	20 (38.4%)		
	Flexion [n (%)]	32 (30.8%)	16 (30.8%)	16 (30.8%)		

*$p < 0.05$; bCP, bilateral cerebral palsy; uCP, unilateral cerebral palsy; MWU, Mann-Whitney U test; χ^2, Pearson chi squared; GMFCS, gross motor function classification system; GPS, gait profile score; GVS, gait variable score; IQR, interquartile range.

Table S5. Pearson chi-squared associations between the thalamus and GMFCS in children with uCP.

		Thalamus	
		**0**	**1**
χ^2	4.13[a]*		
**V**	0.282[b]		
**ASRs**			
**GMFCS I**		2.0	-2.0
**GMFCS II**		-2.0	2.0

*p ≤ 0.05; GMFCS, gross motor function classification system; uCP, unilateral cerebral palsy; χ^2, Pearson chi squared; V, Cramer's V, indicating a significantly weak association based on the degrees of freedom (DF) explained in Supplementary Table S1 [2]; ASRs, adjusted standardized residuals; [a]results should be interpreted with caution because >20% of cells had expected frequencies lower than n=5; [b]DF=1.

Table S6. Pearson chi-squared associations between brain lesion extent scores and the multiple joint patterns in the total, bCP and uCP groups.

			Corpus callosum[a]			
			**0**	**1**	**2**	**3**
**Total**	χ^2	15.20*				
	**V**	0.303[c]				
	**ASRs**					
	Minor				2.3	-2.0
	Extension		2.3		-2.9	
	Flexion		-2.4			
**uCP**	χ^2	18.51[b]**				
	**V**	0.404[c]				
	**ASRs**					
	Minor					
	Extension		2.2		-3.0	
	Flexion					2.5

*p ≤ 0.05; **p ≤ 0.01; bCP, bilateral cerebral palsy; uCP, unilateral cerebral palsy; χ^2, Pearson chi squared; V, Cramer's V, indicating significantly moderate (gray) and stronger (darker gray) associations based on the degrees of freedom (DF) explained in Supplementary Table S1 [2]; ASRs, adjusted standardized residuals; PV, Periventricular; [a]n_{total}=83, n_{bCP}=38, n_{uCP}=45; [b]results should be interpreted with caution because >20% of cells had expected frequencies lower than n=5; [c] DF=2.

Table S7. Pearson chi-squared associations between brain lesion location scores and the multiple joint patterns in the total, bCP and uCP groups.

		Frontal lobe									PV layer									Anterior Corpus callosum[a]		Middle Corpus callosum[a]	
		**0**	**0.5**	**1**	**1.5**	**2**	**2.5**	**3**	**3.5**	**4**	**0**	**0.5**	**1**	**1.5**	**2**	**2.5**	**3**	**3.5**	**4**	**0**	**1**	**0**	**1**
**Total**	χ^2										32.56[b]**												
	**V**										0.396[c]												
	**ASRs**																						
	Minor													2.7					-2.3				
	Extension														-2.7				2.3				
	Flexion														2.3								
**bCP**	χ^2										23.74[b]*												
	**V**										0.478[c]												
	**ASRs**																						
	Minor													2.8									
	Extension														-2.5				2.2				
	Flexion														2.5								
**uCP**	χ^2	25.09[b]*																		6.78[b]*		12.47**	
	**V**	0.491[c]																		0.388[d]		0.526[d]	
	**ASRs**																						
	Minor		2.0	-2.6			2.2																
	Extension			3.0	-2.6																	3.0	-3.0
	Flexion																			-2.5	2.5	-3.1	3.1

*p ≤ 0.05; **p ≤ 0.01; bCP, bilateral cerebral palsy; uCP, unilateral cerebral palsy; PV, Periventricular; χ^2, Pearson chi squared; V, Cramer's V, indicating significantly moderate (gray) and stronger (darker gray) associations based on the degrees of freedom (DF) explained in Supplementary Table S1 [2]; ASRs, adjusted standardized residuals; [a]n_{total}=83, n_{bCP}=38, n_{uCP}=45; [b]results should be interpreted with caution because >20% of cells had expected frequencies lower than n=5; [c] DF=2; [d] DF=1.

References

1. Fiori S, Cioni G, Klingels K, Ortibus E, Van Gestel L, Rose S, et al. Reliability of a novel, semi-quantitative scale for classification of structural brain magnetic resonance imaging in children with cerebral palsy. Dev Med Child Neurol. 2014 Sep;56(9):839–45.

2. Cohen J. Statistical power and analysis for the behavioral sciences (2nd ed.). Hillsdale, Erlbaum; 1988.

3. Papageorgiou E, Simon-Martinez C, Molenaers G, Ortibus E, Van Campenhout A, Desloovere K. Are spasticity, weakness, selectivity, and passive range of motion related to gait deviations in children with spastic cerebral palsy? A statistical parametric mapping study. PLoS One. 2019;14(10).

4. Himmelmann K, Horber V, De La Cruz J, Horridge K, Mejaski-Bosnjak V, Hollody K, et al. MRI classification system (MRICS) for children with cerebral palsy: development, reliability, and recommendations. Dev Med Child Neurol. 2016;1–8.
